# Comparative effectiveness research: what to do when experts disagree about risks

**DOI:** 10.1186/s12910-017-0202-0

**Published:** 2017-06-19

**Authors:** Reidar K. Lie, Francis K.L. Chan, Christine Grady, Vincent H. Ng, David Wendler

**Affiliations:** 1 Department of Philosophy, University of Bergen, Sydnesplassen 12, 5020 Bergen, Norway; 2 Faculty of Medicine, The Chinese University of Hong Kong, Hong Kong Special Administration Region, People’s Republic of China; 3 Department of Bioethics, National Institutes of Health, 10 Center Drive, Bethesda, MD 20892 USA; 4 Department of Mathematics and Science, Northern Virginia Community College, Woodbridge, VA 22191 USA

**Keywords:** Standard of care, Research ethics, Risk judgments, Research ethics review, Equipoise

## Abstract

**Background:**

Ethical issues related to *comparative effectiveness research*, or *research that compares existing standards of care*, have recently received considerable attention. In this paper we focus on how Ethics Review Committees (ERCs) should evaluate *the risks* of comparative effectiveness research.

**Main text:**

We discuss what has been a prominent focus in the debate about comparative effectiveness research, namely that it is justified when “nothing is known” about the comparative effectiveness of the available alternatives. We argue that this focus may be misleading. Rather, we should focus on the fact that some experts believe that the evidence points in favor of one intervention, whereas other experts believe that the evidence favors the alternative(s). We will then introduce a case that illustrates this point, and based on that, discuss how ERCs should deal with such cases of expert disagreement.

**Conclusion:**

We argue that ERCs have a duty to assess the range of expert opinions and based on that assessment arrive at a risk judgment about the study under consideration. We also argue that assessment of expert disagreement is important for the assignment of risk level to a clinical trial: what is the basis for expert opinions, how strong is the evidence appealed to by various experts, and how can clinical trial monitoring affect the possible increased risk of clinical trial participation.

## Background

Ethical issues related to *comparative effectiveness research*, that is, *research that compares existing standards of care*, have recently received considerable attention. Such research is warranted when the evidence is insufficient to decide between alternate interventions that different clinicians may reasonably offer their patients. Comparative effectiveness research is done with the hope that additional research will shed light on whether one option is superior or causes fewer adverse effects.

In this paper we focus on how Ethics Review Committees (ERCs) should evaluate *the risks* of comparative effectiveness research. In cases of insufficient evidence to make a definite judgment, reasonable physicians and patients may prefer a particular intervention, at the same time as they recognize that other patients and physicians prefer alternative interventions. The reason for the disagreement is not, in these cases, because the two alternative interventions are known to have different risk or benefit profiles, but, in the absence of a universally agreed upon standard for evaluating evidence, experts disagree about what the probabilities of the risks and benefits of the alternative interventions *are*. The challenge for ERCs in these cases is how they should assess conflicting judgments by relevant experts. We shall introduce a case that illustrates this challenge.

First, however, we discuss what has been a prominent focus in the recent debate about comparative effectiveness research, namely that it is justified when “nothing is known” about the comparative effectiveness of the available alternatives. We argue that this focus, exemplified by the controversy over the SUPPORT trial, may be misleading. Rather, we should pay more attention to the fact that some experts believe that the evidence points in favor of one intervention, whereas other experts believe that the evidence favors the alternative(s). We will then introduce a case that illustrates this point, and based on that, discuss how ERCs should deal with such cases of expert disagreement. Our case involves two options to prevent cardiovascular events, both of which have a risk of serious bleeding, but the experts disagree about the probabilities of bleeding. We argue that ERCs have an obligation to ensure that they have assessed the range of expert opinions when they identify research risks and make decisions about the appropriateness of research. We will begin by briefly reviewing the controversy over the SUPPORT trial.

### The SUPPORT trial

The US regulatory agency, the *DHHS Office for Human Research Protection,* raised concerns about a case of comparative effectiveness research in a very public way when they sent a letter in 2013 to the University of Alabama regarding the SUPPORT (Surfactant, Positive Pressure, and Pulse Oximetry Randomized Trial) study, published in the *New England Journal of Medicine* in 2010 [1]. In this trial, 1316 premature infants were randomized into two groups, with different oxygen saturation targets, in one between 85% - 89%, and the other 91% – 95%. Both target ranges were reported to be within the clinically used and accepted limit. Evidence suggested that O_2_ saturation levels higher than the target range may improve overall survival of infants but may increase the chance of oxygen-induced retinopathies in infants resulting in blindness. The target range studied in the trial reflected a tradeoff between maximizing survival and minimizing adverse events, such as retinopathies, at the lower end. At the start of the trial, it was argued that there was insufficient evidence of any survival benefit among infants who would get the higher end of O_2_ saturation, in fact all evidence showed that there was *no* difference in survival within the range provided in the trial [2]. It was also not clear there would be any benefits in terms of reduction in retinopathy at the lower end of the acceptable target range. The investigators argued that since all the infants in the trial received the standard of care- i.e. clinically acceptable and standardly utilized O_2_ saturation levels-, there was no additional risk to these infants by participating in this research project.

The OHRP disagreed with this assessment. They asserted that this study was more than a minimal risk study, and that the disclosure of the risks of the study in the informed consent form was inadequate. In fact, in their letter OHRP describes this study as one that involves *substantial risks* to the infants [3]. This determination caused an extensive debate in the bioethics literature, with prominent bioethicists both supporting [4] and disagreeing [5] with OHRP’s assessment of the risks.

### The lack of attention to expert disagreement

There is a straightforward argument for why research is minimal risk when it compares two interventions that are used and recommended by competent physicians in their ordinary clinical care of patients. The argument applies whenever there is no evidence that would lead reasonable clinicians to recommend or reasonable patients to prefer one or the other intervention. Although some existing evidence might favor one intervention, and other evidence favor the alternatives, there is no evidence that picks out *one* intervention as clearly superior. Nevertheless, for a variety of reasons, some physicians and patients prefer one of them, resulting in variations in what is actually provided in ordinary, clinical care. These ‘reasons’ could be experience, ‘gut feelings’ or ‘institutional policies’ based on a particular adverse event in that institution, or something else. Despite such personal reasons for preferring one intervention, these are not necessarily reasons for others to prefer that same intervention. If all reasonable people agree that there is no knowledge that would lead one to definitively recommend one or the other intervention, then allocation to one or the other treatment arms in a trial is no more and no less reasonable than the allocation in actual practice, which is based on factors that are just as random as the formal randomization procedure in the trial. Hence, there is no added risk of trial participation. Of course it may turn out that one option is shown to be better than another in the trial, but at the time of trial initiation there is no basis for saying that trial participation adds any risk compared with choosing one of the options outside the trial context. [2, 6].

This argument is clearly stated by Lantos et al. in a discussion of a trial comparing two doses of aspirin to prevent cardiovascular events [6]. They argue that such a study is minimal risk

because the patients would be facing those risks anyway, and *it is unknown* [our emphasis] whether participation will increase, decrease, or have no effect on the risk levels. Thus there is no reason to believe that being in the study is any riskier than those patients’ daily lives. While random treatment assignment will shift the treatments given to some patients within the range of currently accepted standards of care, given the uncertainty about which treatment is superior (which is the essential presupposition of clinical trials), *no patient or physician* [our emphasis] would be sure whether this random shift in treatment would lead to benefit or harm for either group of patients and thus for any individual patient within those groups. We believe that the SUPPORT study occurred in precisely this set of circumstances.

In this quotation the connection is made between “not knowing” and judgments by *all* physicians and patients that they are uncertain about which intervention is superior. The statement “it is unknown”, is therefore understood as “all experts agree that it is unknown” or “all experts agree that there is no evidence for the superiority of one intervention over another”.

Critics of the SUPPORT study made a number of arguments, all of which essentially question whether nothing is known about the consequences of different oxygen levels, or that there is agreement about this among experts. They pointed out that in research one cannot individualize treatment, or that treatment assignment in research is according to protocol which is different from ordinary clinical care, where one may change treatment according to a patient’s individual characteristics [7]. But this only changes the assessment of the risk of the research if some experts believe that changing oxygen levels individually makes a difference. If nobody knows whether assignment to a particular point within a range is more or less risky, and this is true for both the initial setting and for any changes that might occur over the course of the trial, the method of assignment cannot affect the prospective risk assessment, and there is no reason to believe that assignment based on certain individual patient characteristics is more or less risky than assignment according to a fixed protocol. But if claims about the benefits of individualization are contentious among experts, then a case can be made that trial participation may have a different risk profile.

Similarly, Silverman and Dreyfuss made a general argument that since clinical trial participants in the SUPPORT trial received care within restricted ranges of that provided in the ordinary clinical care context, they did not receive the same standard of care as those who did not participate in the trial [8]. They quote with approval from the OHRP determination letter. Altering the range of oxygen levels an infant was supposed to receive was a crucial part of the study design. By creating two groups receiving two discrete ranges of oxygen levels, the study increased the likelihood that there would be significant differences in outcomes observed between the two groups.

This argument is only valid if there is reason to believe that restricting the range to two discrete ranges will affect the risk to the subjects. But if all experts agree that based on available evidence, there is no reason to believe that any particular sub-range is better than another, then there is no basis for such a claim.

The argument put forward by Lantos et al. therefore depends crucially on what is the best characterization of the state of knowledge before a trial is initiated. If everyone agrees, meaning that there is consensus among experts that there is no evidence for the superiority of one intervention over another, then randomization to eligible interventions does not increase risk. But if there is expert disagreement about the overall benefits and risks of eligible interventions, then randomization may not be risk neutral. Kim and Miller have indeed emphasized the varieties of standard-of care-research [9]. In certain cases, some experts argue that evidence points in one direction, others argue that evidence points in another direction. Some experts will conclude that, given the evidence, one intervention is to be preferred over the others, others will recommend alternatives. While the premise accepted by Lantos et al. may be true for some trials, and it may be true for the SUPPORT trial in particular, it does not follow that it is true for a large number of comparative effectiveness trials. In particular, there is a range of cases about which the experts disagree about what the evidence says about the benefits and risks of the various interventions in use. Lantos himself may have recognized this point when he said in reply to criticism of his position:

These post-SUPPORT controversies suggest the complexities that we face going forward. If experts cannot agree about how to quantify the risks and benefits of a study even after the results are available, it is hard to imagine how we will ever agree on an appropriate way to describe the risks and benefits of studies before the results are known. Solving this problem will be more important than coming to consensus about the specific issues of SUPPORT [10].

We therefore need to pay more attention to expert disagreement when assessing clinical research. Cases of expert disagreement present tough challenges for an ERC when they need to assess risk-benefit profiles of research. We shall now present a case that illustrates this point.

## Main text

### The case of antiplatelet therapy to prevent cardiovascular risk

Antiplatelet therapy has long been recognized as an important part of a treatment and prevention program in patients with coronary artery disease. In 2000, for example, the American College of Cardiology/American Heart Association made the following recommendation:

“Antiplatelet therapy should be initiated promptly. Aspirin (ASA) is the first choice and is administered as soon as possible after presentation and continued indefinitely”.

The major concern with administering aspirin is the risk of gastrointestinal bleeding. This is particularly the case for patients who have had a history of such bleeding. Therefore, the ACC/AHA guidelines recommended in 2000 that

“A thienopyridine (clopidogrel or ticlopidine) should be administered to patients who are unable to take ASA because of hypersensitivity or major gastrointestinal intolerance”[11].

The guideline classified the strength of evidence for both of these recommendations. The first is a class 1 A recommendation, and the second is a class 1 B recommendation. Class 1 means ‘general agreement that a given procedure is useful and effective”. The addition of A means that there are “data from multiple randomized clinical trials that involved large numbers of patients”, and B means that “data were derived from a limited number of randomized trials that involved small number of patients or from careful analyses of nonrandomized studies or observational registries”. In 2002 the guideline was updated and changed slightly. Only clopidogrel was recommended for patients who cannot take ASA, and the strength of evidence for this recommendation was upgraded to 1A [12].

Some experts disagreed with the evidence that supported this opinion. This was especially true for experts in the prevention of gastrointestinal bleeding, while the recommendations in favor of clopidrogel originated within the community of cardiovascular clinicians. In 2005 Chan et al. published a study where they compared clopidogrel alone to aspirin together with a proton pump inhibitor [13]. Patients who were on a preventive aspirin regimen and who experienced a bleeding ulcer were randomized to one of the two intervention arms, after their ulcer was healed. They showed that aspirin together with a proton pump inhibitor (PPI) was far superior to clopidogrel alone in the prevention of recurrent upper gastrointestinal bleeding. Another trial published in 2006 confirmed this finding. In 2007 the revised ACC/AHA guidelines recommended, based on the 2005 study by Chan et al., that a proton pump inhibitor be added to clopridogel in patients with a history of gastrointestinal bleeding with aspirin [14], even though Chan et al.’s study had only demonstrated the superiority of ASA plus PPI over clopidogrel alone. Finally, in 2008 a consensus document was published on reducing the gastrointestinal risks of antiplatelet use. The document stated that “Substitution of clopidogrel for ASA is not a recommended strategy to reduce the risk of recurrent ulcer bleeding in high risk patients and is inferior to the combination of ASA plus PPI.” [15].

The case illustrates both the dilemma and the importance of doing clinical research on existing interventions. On the one hand, in 2002 there were clear and unambiguous recommendations by reputable professional societies, recommending clopidogrel alone in patients at risk for gastrointestinal bleeding. Based on that recommendation, the proposed trial proposed by Chan et al. would expose half of the trial population to unacceptable risks. Experts who supported the 2000/2002 ACC/AHA guidelines did actually criticize the trial by Chan et at, noting that they were “disturbed by the scientific rationale and questionable design of the current trial” [16]. On the other hand, there were reasonable doubts about the soundness of the existing recommendations. An appropriate clinical trial would settle the issue, but ERCs and experts may be reluctant to approve it given the strong recommendations in favor of one intervention.

This is clearly *not* a case where experts *agreed* that there was insufficient evidence to distinguish between the two interventions in the trial. Some experts believed that the evidence identified one intervention as clearly superior, even classifying the strength of evidence as 1A. However, other experts believed that the evidence supported a different approach. How should we evaluate the risk of trial participation in cases such as this one?

Emily Evans and Alex London have previously argued for the importance of making a distinction between what they call cases of *agnosticism* and cases of expert disagreement when deciding about enrolling human subjects for research [17]. They define agnosticism as a state where “members of the expert medical community have not yet made determinate judgments about the relative therapeutic merits of a set of interventions”. They apply this to the policy level about what intervention to recommend for groups of patients, and argue that it is consistent with individual physicians making more specific recommendations for individual patients. Examples would be if one cannot make a general policy recommendation for a particular intervention for all patients, but some physicians recommend one specific intervention for older patients. We have identified a similar state where there is consensus among experts that there is no evidence that would favor one intervention over the other, but this would apply both at a policy level and for individual recommendations: the focus is on the agreement that not enough is known, but it can apply both to groups and to individuals. Evans and London, and we, contrast this with a state where there is disagreement among relevant experts about the relative therapeutic merits of a set of interventions. Again, we focus on the policy or general level about what to do for classes of patients, whereas for Evans and London the focus is on disagreements about individual patients. They argue that in cases of such disagreement there is clinical equipoise and entering a particular patient to a trial is admissible:

If at least a reasonable minority of experts would recommend A over B as treatment for C, while others recommend B over A, then we hold the option of random assignment [of a particular patient] to a trial of either A or B admissible.

This assessment about an individual subject takes place after an ERC has approved the trial, and decided that the risks to the subjects are reasonable in relation to anticipated benefits. Before starting to enroll individual subjects, therefore, clinical researchers and ERCs must decide whether a particular trial is justified at all in terms of the potential risks to subjects. While the decision rule proposed by Evans and London may apply when experts disagree about what to do for individual patients, it is difficult to see how one could apply it when deciding what the risk of trial participation is for all eligible subjects in a proposed clinical trial, hence, difficult to see how it helps ERCs to decide whether a trial should be approved. This is the decision faced by ERCs, research sponsors and researchers themselves. We shall now address the complexities of such risk assessments when experts disagree.

### ERCs and risk assessments

A common challenge of comparative effectiveness research is therefore how an ERC should evaluate the risks of a trial when experts disagree in the sense of endorsing different approaches. An ERC needs to provide an overall risk assessment when experts disagree, taking the interests of all prospective research subjects into account. In some cases, such as when dealing with participants who cannot give consent themselves, ERCs are also required to provide an explicit risk assessment of the research, to ensure that it does not exceed the risk threshold above which the research cannot be approved.

It seems that an ERC has essentially three options, all of which are unsatisfactory. They can accept the risk judgments of one group of experts over the other, they can make their own assessment of the risk level of the individual interventions, or they can conclude that it is impossible to arrive at any definite judgment about risk. The two first options presuppose that members of ERCs have sufficient expertise to make their own risk judgments, which they typically do not have. The third option would mean an end to a lot of comparative effectiveness research. There is, however, a fourth option which does not involve making judgments about what the risks of the individual options *are* or about how reliable the evidence for each of the options actually *is*. We shall now present this fourth option.

### How ERCs can make risk judgments when experts disagree

When deciding what the risk level of a trial is, the ERC need not, and should not, accept the risk judgment of one particular group of experts, nor should they make an independent assessment of the evidence, and make their own risk judgment. Instead, they should assess the range of expert opinion and base their judgment on *that* assessment. If experts agree, an ERC should use that agreement as a basis for their judgment. In the special case when experts agree that *it is unknown* whether one intervention is riskier than others, i.e. all experts agree with that assessment, then that is what the ERC should base their risk assessment on. However, if there is disagreement about the evidence among reasonable experts, the ERC should make a decision on that basis.

It is important to note that in the following we assume that disagreements among recognized experts are genuine. By genuine disagreements we mean that there is some evidence, recognized by all, in favor of one intervention, and some evidence in favor of other interventions. The disagreement is about the weighting of the evidence, and what the evidence in favor of the interventions is, all things considered. Trials should aim to respond to these disagreements by gathering additional evidence about the interventions in comparison to each other. By recognized experts we mean professional societies, or groups of researchers at leading institutions. This is meant to exclude, in this context, individual researchers who hold idiosyncratic opinions, or appeal to evidence that is not generally accepted. In particular, an ERC should not assume, without further argument, that the researchers submitting a protocol for review, although they may be experts, fulfill our criterion of recognized experts.

One might worry that expert disagreements can lead to decision paralysis for ERCs: how can an ERC arrive at a decision when no matter what they decide, there will always be reputable experts who disagree with them? In the clopidogrel case, for example, those who adhered to the practice guidelines would have criticized the ERC if they approved the trial, because they believed that it exposes subjects to unacceptable risks. If they do not approve the trial, the ERC would have been criticized by other, equally reputable experts, because they are not willing to approve comparative investigation of unproven interventions.

In order to see that it is possible to approve trials even when experts disagree in this way, let us begin by assuming a simple case where there are two experts, and two interventions, and first assess the risks a patient faces making a clinical choice. For each intervention, the combined frequency/probability of adverse events is given, and they are equally severe in the two groups, but the probabilities differ. We also assume that there is no uncertainty in the estimate of these probabilities, according to the individual expert assessments. The two experts may disagree with each other about the uncertainties, but are confident of their own risk assessment. The positive effects of the interventions are the same for both interventions. Given the disagreement between the two experts about the risks of these interventions, what would a rational patient choose? In order to answer that question, we need to distinguish between four different scenarios. Consider the scenario provided in Table 1. According to expert 1, intervention A has a combined risk frequency of 1%, whereas intervention B has a combined risk frequency of 2%. Expert 2 has exactly the opposite assessment. In this first scenario, there seems to be no basis for preferring one particular intervention. If expert 1 is right, then intervention A is the preferred choice, with an expected frequency of side effects of 1%. But if expert 2 is right, the expected frequency is 2%. Intervention B has exactly the same expected side effects, but associated with the opposite interventions according to the experts. In this case there is no reason to prefer one of the interventions to the other.

**Table 1:** Two experts with opposite assessments of risks

	Expert 1	Expert 2
Intervention A	1%	2%
Intervention B	2%	1%

In our scenarios we have used a standard decision theoretic framework with two alternative actions, but the uncertain outcomes are states of affairs that occur if one of two experts are correct in their assessments, and the probability that each group of experts is correct is the same, i.e. 50% in our example. The utilities associated with these states are the same in all possible outcomes, but may occur with different known probabilities according to the experts. Since we are considering side effects, the outcomes are better the lower the probabilities of the side effects.

In the second scenario (Table 2), there is a reason to choose intervention A. The best outcome would be 1% independent of which expert is correct. The worst outcome would be 3% if expert 1 is correct but 2% if expert 2 is correct. In the third scenario (Table 3), there seems to be a reason to choose intervention B. The worst outcome is 2%, but the best outcome is 0.5% if intervention B is chosen, compared with 1% if intervention A is chosen.

**Table 2:** One outcome is best independent of expert disagreement

	Expert 1	Expert 2
Intervention A	1%	2%
Intervention B	3%	1%

**Table 3:** Experts agree on worse outcome, but one expert favor one intervention

	Expert 1	Expert 2
Intervention A	1%	2%
Intervention B	2%	0.5%

The fourth scenario (Table 4) is more difficult. Here it would depend on how risk averse the patient is. The expected benefit is higher for intervention B, but the worst case scenario is worse for intervention A.

**Table 4:** Choice depends on risk aversion

	Expert 1	Expert 2
Intervention A	0.5%	3%
Intervention B	2%	1%

If a clinical trial is proposed to compare interventions A and B, it would be uncontroversial in the first scenario, but problematic, given the assumptions, in the three other scenarios. The assumptions are that we have no reason to prefer one group of experts to another, that there is no uncertainty about the probability estimates within the expert group, and the values of the risk are the same in all outcomes. We will return to a discussion of how an ERC should consider the risks of trials in these other scenarios after we have applied our framework to the clopidogrel case.

The proposed trial compares two interventions that were both available when Chan et al. started their research in the early 2000s. The rationale was, in the opinion of the investigators, that there was little justification for the clinical guidelines in place at the time, specifically that clopidogrel had fewer gastrointestinal side-effects compared with aspirin. Many disagreed with this assessment, as is reflected in the guidelines. Practice guidelines are one important source for the identification of standard of care. The 2002 guideline clearly stated that the standard of care for patients with high risk of gastrointestinal bleeding who need to be on an antiplatelet preventive regimen was clopidogrel. However, the justification for the guideline was less clear, especially for giving clopidogrel class 1A recommendation status. This recommendation seems to be based on one clinical trial comparing clopidogrel with aspirin, and the aim of this trial was not to assess the advantages of using clopidogrel in patients with a history of gastrointestinal bleeding [18], but rather as a secondary outcome, a reduction in gastrointestinal bleeding was associated with clopidogrel use. In fact, therefore, there was sparse direct clinical trial evidence that clopidogrel was the preferred preventive regiment in this patient group. There was some evidence of effectiveness from a clinical trial, but definitely not “data from multiple clinical trials that involve large numbers of patients”. Clopidogrel’s pharmacological properties also suggested it would cause less bleeding risk than aspirin, but such inferences are known to be unreliable in the absence of hard clinical trial evidence. In the absence of conclusive evidence that clopidogrel could indeed lower overall risks of gastric bleeding, reasonable clinicians had legitimate reasons to question the guideline and disregard this advice, as has been discussed elsewhere [19]. Reasonable clinicians therefore argued that since aspirin is actually the preferred preventive strategy in the guideline, this drug should be prescribed to patients with high risk of bleeding together with a proton pump inhibitor (PPI) that is known to causally block the formation of bleeding ulcers.

There were therefore two groups of experts, one group represented by the clinical practice guidelines who claimed that clopidogrel had fewer gastrointestinal side-effects than aspirin, and the other group who claimed that there was reason to believe that aspirin together with a PPI would have fewer side-effects than clopidogrel alone. If we assume that the two regimens have the same positive effects in preventing cardiovascular events, this is a case similar to scenario 1 above. In spite of expert disagreements, a rational patient or provider had no definitive reason to prefer either one of the two interventions outside of the trial. If there is no reason to prefer either one of the two interventions, there is also no reason to prefer a choice of one of the two over randomization to one of the two interventions, given our assumptions.

### The risks of therapy and the risks of research

The clopidogrel case turns out to be relatively straightforward given our assumptions because the choice faced by patients in the clinical setting was essentially no different than choosing between entering a clinical trial or choosing one of the interventions outside the trial even though the overall aggregate risks might have turned out to be very different once the trial was completed. In this sense this case is structurally similar to cases where there is agreement among experts that differences between two interventions are unknown. Our focus on the importance of expert disagreement, however, has identified important issues that need to be addressed when ERCs evaluate research when experts disagree about the risks.

First, we have identified three additional scenarios above. The first scenario is a special case because the experts agree on the magnitude of the probabilities; the only disagreement is about which intervention has the higher risk. In the three other scenarios, the magnitudes are also different, and it is then not obvious that a clinical trial can be justified, certainly not as a minimal risk trial, unless the side effects are very minor. It is only in the first scenario that the risk of individual choice of therapy is the same as the risk of research.

Second, we have assumed that there is only one type of risk, in our case gastro-intestinal bleeding, and it is the same for both interventions. Typically, there are several possible adverse events associated with interventions, and they may occur with different probabilities. These adverse events may also be evaluated differently by different people. While it may be possible to combine these into one outcome measure for individuals, it is difficult to see how that can be done on a population level. For many individuals, some outcomes are more important than others, and disagreement about the probabilities for these outcomes are more important than disagreements about other outcomes. If experts disagree about outcomes that are evaluated differently by different patient groups, making risk assessments of trials are going to be much more difficult.

Third, individual attitudes towards risk aversion may also influence choice. This also complicates risk assessments of trials, as was illustrated in scenario 4.

Fourth, we have assumed that there is no uncertainty associated with the probability judgments within the individual expert groups.

While some of these assumptions may make it more difficult to justify a trial, because the trial is more risky than ordinary clinical care, even when experts disagree, others actually make it possible for the trial to be less risky than ordinary clinical care. The relevant risk assessment for the ERC is not exactly the same as that faced by the clinical patient. The ERC needs to compare the risks patients face when making decisions about therapeutic choices in situations where experts disagree about the consequences of the options, with the risks they face in clinical trials where experts disagree about options that are provided by a random mechanism: What are the overall risks of ordinary therapeutic choices compared with the overall risks of random assignment to the relevant therapeutic options? There are two ways in which the risks of the trial may be reduced compared with ordinary clinical care.

First, one may restrict the selection of subjects to a particular group, based on their preferences, either by explicit inclusion and exclusion criteria, or by self-selection based on information provide to prospective participants. Decisions about criteria used would depend crucially on a proper analysis of expert views and where they disagree. Basically, the aim would be to change scenarios 2–4 to scenario 1, by excluding subjects with certain types of risk, or certain types of evaluations of risks, or with a particular risk aversion profile.

Second, one may introduce risk reduction strategies as part of the clinical trial itself, such as increased monitoring. The aim would again be to make the trial conform to the first scenario, by concentrating on reducing the risk levels for those events where there is disagreement about the risk levels among the experts (scenarios 2 & 3 above). Receiving any treatment in a clinical trial setting may be less risky than receiving the same treatment outside the clinical trial setting, because of increased monitoring, more careful follow up, or other factors. For example, in the clopidogrel trial extra monitoring within the trial might identify bleeding and allow for treatment sooner than in standard clinical care.

### Obligations of ERCs

Our analysis of how to assess the risks of comparative effectiveness research in situations where experts disagree has focused on how ERCs should assess the risks of such research. One may object to our analysis by pointing out that assessing the risks of research should be the responsibility of the researchers themselves, and placing this responsibility on ERCs will place additional burdens on committees that already have difficulties fulfilling their mandated tasks within the resources allocated to them.

It is important to note that we do not recommend that ERCs should take on the responsibility of evaluating the scientific evidence, or do formal literature reviews. In fact, we explicitly reject this option above. Assessing the relevant science for a clinical trial is the responsibility of the proposing researchers and sponsors of such research, and is routinely done in protocols submitted to ERCs. ERCs, however, do have a responsibility to ensure that this assessment is unbiased in the sense that all relevant expert opinions have been included in the assessment. ERCs cannot simply rely on a particular group of experts for their decision, but they need to do a systematic review of the range of expert opinions about the interventions in the proposed study. We do not propose that ERCs do a systematic review of the actual evidence, but rather a review of relevant expert assessments.

In the case introduced here it would have been wrong for the ERC to simply base their decision on the practice guideline adopted by American College of Cardiology/American Heart Association, which was what the ERC did when they did not at first approve the Chan et al. 2005 study. It would have been equally wrong for the ERC to simply base their risk assessment on the views of experts who supported the 2005 study, ignoring the ACC/AHA guideline, in particular because the investigators were from their own institution, and even though they turned out to be correct in their assessment of the risks. An ERC has an obligation to identify the range of expert opinion, and decide based on their own assessment of expert opinion, along the lines suggested above. The ERC may very well agree that this study is minimal risk, but not because all experts agree that one intervention is no better than the other. The ERC could decide that the study is minimal risk because based on their assessment of expert opinions, it concluded that patients in the trial are not exposed to a higher level of risk than those choosing their preferred intervention outside of the trial. In other words, overall there is no reason for an individual patient to prefer either A or B. Hence the study can be categorized as minimal risk even though all the experts believe the risks of one of the study interventions are greater than the risks of the other study intervention.

It follows from this that ERCs need to do a much more thorough review of the risks of research than is typical today. They need to be confident that they have assessed all relevant views and doubts about standards of care. It is not enough to simply point out that the interventions provided in the trial are also provided outside the trial, or that a particular professional society has issued a guideline or recommendation. Table 5 summarizes the types of issues an ERC needs to assess (domains), and examples of what the ERC needs to do (details). It is important to note that the emphasis in this table is on the responsibility of the ERC to identify relevant expert opinions and to assess the relevance of any disagreements among experts, but it continues to have to rely on experts, including the researchers and sponsors who have submitted the trial for review, for the scientific review of existing evidence. However, the ERC should not simply register expert disagreement, but need to assess the nature and the scope of such disagreements. Not all expert disagreements have the same implications for ERC risk assessments.

**Table 5:** List is issues that need to be addressed by ERCs

Domain	Details
Identify relevant experts	- Include recognized experts
	- Exclude experts with obvious bias
Identify relevant outcomes and associated values	- Identify range of values associated with the various outcomes among relevant subject groups
Identify probabilities of outcomes	- Identify relevant disagreements about probabilities among expert groups
	- Identify uncertainty about probability estimates within expert groups
Identify risk of research compared with clinical care	- What is the structure of the choice between the two interventions given expert disagreement
Identify possible risk reduction strategies	- Modify selection of subjects
	- Introduce risk reduction strategies in the trial

## Conclusions

The issue of how to assess the risks of comparative effectiveness, or standard of care, research, has been debated lately. In this paper we have argued that it is necessary to distinguish cases where there is consensus among experts that there is insufficient evidence favoring one or the other standard interventions from cases where experts disagree about the evidence. When experts disagree, we have argued that it necessary to assess the nature of the disagreement so that the ERC can conclude what the risk level of a study is. In our discussion we have argued that ERCs have a duty to assess the range of expert opinions and based on that assessment arrive at a risk judgment about the study under consideration. We have also argued that assessment of expert disagreement is important for the assignment of risk level to a clinical trial: what is the basis for expert opinions, how strong is the evidence appealed to by various experts, and how can clinical trial monitoring affect the possible increased risk of clinical trial participation. The fact that there is disagreement among experts about which of the alternatives is preferable is not sufficient to conclude that a clinical trial is acceptable or poses minimal risk. The review committee needs to further evaluate whether the disagreements between experts essentially cancel each other out so that there is no reason for an individual patient or provider to prefer either A or B. This would not be the case for example if some experts think that A is slightly less risky than B whereas other experts believe the B is dramatically more risky than A. ERCs therefore need to review the reasons for expert disagreements carefully and systematically before they conclude that a trial has a specific risk level or approve a trial.

## Abbreviations

ACC: American College of Cardiology
AHA: American Heart Associations
ASA: AcetylSalicylic Acid
DHHS: Department of Health and Human Services
ERC: Ethics Review Committee
OHRP: Office for Human Research Protection
PPI: Proton pump inhibitor
SUPPORT: Surfactant, Positive Pressure, and Pulse Oximetry Randomized Trial
